# Carcinoma of stomach detected by routine transabdominal ultrasound

**DOI:** 10.2349/biij.6.4.e39

**Published:** 2010-10-01

**Authors:** MFE Wong, SFJ Shum, WK Chau, CS Cheng

**Affiliations:** 1 Department of Radiology, Pamela Youde Nethersole Eastern Hospital, Hong Kong; 2 Department of Radiology, Ruttonjee and Tang Shiu Kin Hospitals, Hong Kong

**Keywords:** Limb salvage rate, lower limb vascular disease pattern, diabetics, Asian centre, percutaneous angioplasty

## Abstract

Assessment of the stomach is not commonly included in routine scanning protocol of upper abdominal ultrasound (USG). However, assessment of the stomach in patients presenting with epigastric pain can yield invaluable results. This paper presents, as an illustration, a case of carcinoma of stomach detected by transabdominal ultrasound. The diagnosis is confirmed by subsequent CT, upper endoscopy and operation.

## CASE REPORT

A 56-year-old gentleman with good past health presented with epigastric pain and dysphagia for 1 month. He was referred for an elective ultrasound examination. 

**Figure 1:**
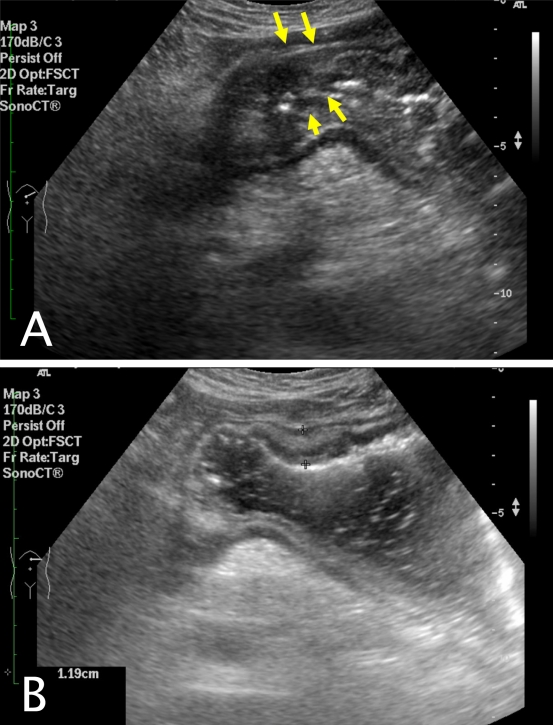
a) Transverse scanning of the upper abdomen of the patient. Nodular and irregular thickening of the gastric antrum with loss of wall stratification (arrows); b) Transverse scan of the upper abdomen after patient takes in water. Wall thickening up to 1.19 cm.

Transabdominal ultrasound showed nodular and irregular wall thickening of the gastric antrum, measuring up to 1.19cm in thickness. There was also associated loss of wall stratification (Figure 1). Small amounts of peritoneal fluid in the left subhepatic space was also seen. Other findings included a small gallbladder polyp and small gallstones. 

**Figure 2:**
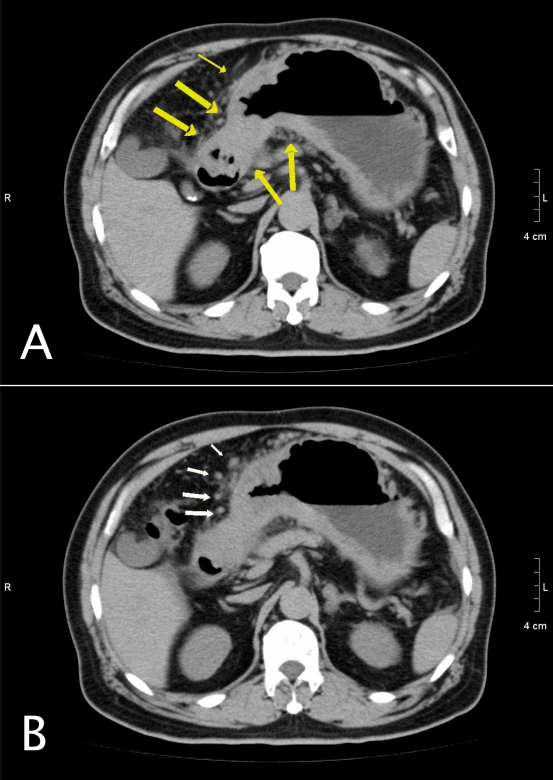
Complementary plain CT of the patient confirms thickening of the stomach wall. Adjacent peritoneal stranding and nodularity (arrows) is seen, which is suggestive of disease invasion.

CT done after the ultrasound examination showed irregular wall thickening in the gastric antrum and body (Figure 2). Adjacent peritoneal fat stranding and nodularity were also present, which is suggestive of disease invasion and lymphadenopathy. 

The patient subsequently underwent elective operation and gastric adenocarcinoma wass confirmed at laparotomy.

## DISCUSSION

**Figure 3:**
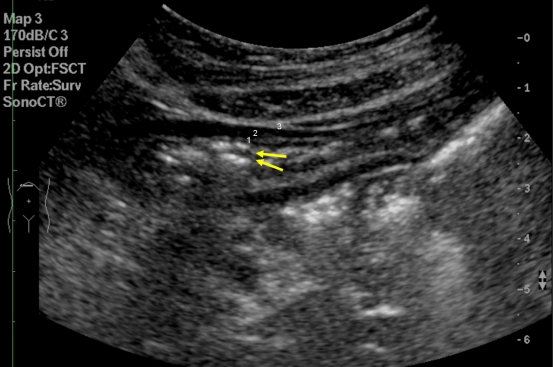
Transverse scan of the upper abdomen of a normal patient. Ring down artifacts (arrows) are suggestive of intraluminal gas in the stomach. Different layers of stomach: 1. echogenic mucousa and submucousa; 2. hypoechoic muscularis propria; and 3. echogenic subserosa.

The normal appearance of the stomach is illustrated in Figure 3. Three distinctive layers can usually be seen in a transabdominal ultrasound. The mucousa and submucousa appears hyperechoic, muscularis propria is hypoechoic and subsererosa is hyperechoic. In endoscopic ultrasound, resolution of the gastric wall into five layers may be seen but that is beyond the scope of this discussion. 

Loss of wall stratification has been shown to be a sign of gastric malignancy [1]. This may be accounted for by the invasiveness of the disease process itself, in which the tumour invades across different layers. Gastric wall thickening without loss of wall stratification favours a benign process, such as gastric ulcer, Menetrier’s disease and anisakiasis.

Apart from loss of wall stratification, the degree of gastric wall thickening also gives a clue to the nature of underlying disorder. The sonographic thickness of normal gastric body and antral wall measures up to 5mm in a non-distended state [2]. Wall thickening of a lesser extent (5-8mm) favours benign causes, such as chronic gastritis and gastric ulcer.[3]. In malignant causes, the degree of thickening is greater, with average thickness reported to be 15.9 mm in a study [4].

In a healthy population, visualisation of the gastric antrum has been reported to be up to 100%, while the body and fundus is less consistently seen [5]. Transabdominal ultrasound examination has also been shown to have high efficacy in visualising gastric carcinoma [6]. Despite these promising results, however, the detection of gastric tumours in real life practice depends on patient habitus, location and staging of the gastric tumour. 

In most centres, routine scanning protocol of the upper abdomen includes the liver, gallbladder, pancreas, kidneys and spleen. Little attention has been paid to the stomach. It is a common belief among sonographers and radiologists that gastric pathology cannot be picked up by ultrasound. Indeed, some part of the gastric wall may be obscured by intraluminal gas. However, ingestion of water just before the examination will help to displace intraluminal gas and provide an acoustic window for visualisation of the posterior wall. 

Another limitation in transabdominal ultrasound is in the detection of early mucousal lesions. Early tumours that have not yet reached the stage of frank submucousal invasion and formed a reasonable tumour bulk may not be readily picked up by transabdominal ultrasound. In such cases, patients with a negative ultrasound finding but with relevant symptoms should not be deferred for endoscopy. However, frank gastric pathology such as the one illustrated in this case study should be picked up during routine ultrasound, which will guide further patient management. 

In summary, the authors recommend that attention be paid to gastric wall pathology during routine scanning for patients with relevant symptoms. 
